# Sexual risk behaviour, marriage and ART: a study of HIV-positive people in Papua New Guinea

**DOI:** 10.1186/1742-6405-10-17

**Published:** 2013-06-27

**Authors:** Wing Young Nicola Man, Angela Kelly, Heather Worth, Andrew Frankland, Patti Shih, Martha Kupul, Thiri Lwin, Agnes Mek, Barbara Kepa, Rebecca Emori, Frances Akuani, Brenda Cangah, Lucy Walizopa, Lawrencia Pirpir, Somu Nosi, Peter M Siba

**Affiliations:** 1 School of Public Health and Community Medicine, UNSW Medicine, University of New South Wales, Sydney, Australia; 2 Faculty of Health Sciences, University of Sydney, Sydney, New South Wales, Australia; 3 Sexual & Reproductive Health Unit, Papua New Guinea Institute of Medical Research, Goroka, Papua New Guinea

**Keywords:** HIV, Papua New Guinea, Sexual Behaviour, Antiretroviral Therapy

## Abstract

**Background:**

The prevention of intimate partner transmission of HIV remains an important component of comprehensive HIV prevention strategies. In this paper we examine the sexual practices of people living with HIV on antiretroviral therapy (ART) in Papua New Guinea (PNG).

**Method:**

In 2008, a total of 374 HIV-positive people over the age of 16 and on ART for more than two weeks were recruited using a non-probability, convenience sampling methodology. This accounted for around 18% of adults on ART at the time. A further 36 people participated in semi-structured interviews. All interviews were thematically analysed using NVivo qualitative data analysis software.

**Results:**

Less than forty per cent (38%) of participants reported having had sexual intercourse in the six months prior to the survey. Marital status was by far the most important factor in determining sexual activity, but consistent condom use during vaginal intercourse with a regular partner was low. Only 46% reported consistent condom use during vaginal intercourse with a regular partner in the last six months, despite 77% of all participants reporting that consistent condom use can prevent HIV transmission. Consistent condom use was lowest amongst married couples and those in seroconcordant relationships. The vast majority (91.8%) of all participants with a regular heterosexual partner had disclosed their status to their partner. Qualitative data reinforced low rates of sexual activity and provided important insights into sexual abstinence and condom use.

**Conclusions:**

Considering the importance of intimate partner transmission of HIV, these results on the sexual practices of people with HIV on ART in PNG suggest that one-dimensional HIV prevention messages focussing solely on condom use fail to account for the current practices and needs of HIV-positive people, especially those who are married and know their partners’ HIV status.

## Background

Papua New Guinea (PNG) reported its first person diagnosed with HIV in 1987. Although more recent national adult prevalence estimates suggest the epidemic is progressing less rapidly than previously feared with less than 1% (0.9% 2009 estimate and 0.8% 2010 estimate) of the adult population currently estimated to be infected [1,2], HIV prevalence in PNG however remains among the highest in the Asia-Pacific Region. PNG’s HIV epidemic has been, and continues to be, heterosexually transmitted with more women than men diagnosed as HIV-positive [2]. The epidemic exhibits substantial geographic heterogeneity, with nearly 90% of diagnoses coming from the seven Highlands provinces, the National Capital District and Morobe Province [2]. It is widely recognised from within PNG and from international agencies that innovative strategies for the treatment and care of people living with HIV and of HIV prevention are urgently needed to address the complex public health issue that HIV poses in a country with unparalleled geographical, linguistic and cultural diversity [3]. PNG is home to more culturally diverse groups and languages than any other country in the world; for example, it is reported that there are more than 800 living languages [4].

Following a global effort towards universal access to life-saving antiretroviral drugs in developing countries, PNG began its first pilot project of ART in 2004. As a result of efforts to universalize access to ART, treatment has now been rolled out throughout PNG. At the time of the study there were around 38 sites offering ART and fewer than 7,000 people (adults and children) had been registered for treatment [1]. By the end of 2010, 78 sites were offering ART in 20 provinces with 11,244 adults and 610 children registered for treatment since 2004. Of these, 71% of adults and 88.2% of children had commenced treatment [2].

At the time of this study CD4 cell count testing was available only at the central laboratory in Port Moresby and in one other site and thus not widely accessible. Since the time of this study, and as a result of the work of the Clinton Health Access Initiative and the Central Public Health Laboratory, CD4 cell count testing, although not available throughout the country as part of routine clinical management, has now become far more accessible. At the time of writing this paper, viral load testing was still not used as part of the clinical management of HIV in PNG.

It is well documented that ART has dramatically altered the lives of people living with HIV in both developed and developing countries. The impacts of ART have been many and one such area is the realm of sexual behaviour. At the time treatment were becoming widely accessible there was a public health concern that treatments would see an increase in sexual activity, specifically a reduction in safe sex among those living with HIV cf.[5-7]. An early meta-analysis of sexual behaviour and ART with studies, largely drawn from United States of America-based studies and of studies exclusively about or including men who have sex with men, concluded that unprotected sexual behaviour was elevated in people who had less concern regarding unsafe sex as a result of the widespread availability of ART [8].

Until recently the majority of research on the sexual behaviours and practices of people on ART were drawn from developed countries and populations such as men who have sex with men [6,7,9-13]. In more recent years there has been an important and growing body of work emerging from developing countries, particularly from sub-Saharan Africa. These studies suggest there has not been a reduction in reported rates of consistent condom use by HIV-positive people on treatment [12,14-20]. These findings suggest that ART has not led to sexual risk compensation (the abandonment of condoms and other changes to sexual behaviour such as increased number of partners) as was previously feared; thus the gains made in HIV prevention appear to be maintained. Indeed, much evidence now suggests that there are high rates of sexual abstinence in developing countries by those on treatment [5,14,17,18,20-24]. In a recent meta-analysis of cross-sectional and longitudinal studies conducted in sub-Saharan Africa, it was found that individuals on ART were significantly less likely to have unprotected sex or to have multiple sexual partners compared with when they were ART-naïve, but there were no significant differences in sexual activity [25]. Drawn from a larger study of the social experiences of people on ART in PNG [26,27], this paper focuses on sexual behaviour and condom use. Furthermore, set in the country with the greatest HIV epidemic in the Pacific, our wider study was the first known behavioural study of people living with HIV (PLHIV) in the region and provides the only published quantitative data on PLHIV sexual behaviour in the region to date.

## Results

Table 1 presents the socio-demographic characteristics for the entire sample and for men and women separately. Of the 374 study participants, 147 (39%) were men and 227 (61%) were women. The majority (72%) of the sample was aged 35 years or younger, with a median age of 30 years, and a range from 16 to 69 years. The men in the study were significantly older than the women, with median ages of 35 and 28 respectively. Three-quarters of the sample reported having never been to school (21%), or been educated up to elementary (9%) or primary school only (43%). Overall, men reported having attained a higher level of education than women with a significantly greater proportion of men having completed secondary or higher education (39%) compared with women (19%). One-fifth of the overall sample (20%) reported that they drink alcohol, and significantly more men drink (27%) compared with women (17%).

**Table 1:** Sample characteristics

	**Male (n = 147)**	**Female (n = 227)**	**Total (N = 374)**	**p (χ**^**2**^**)**^**a**^
**Age in years**^**b**^	35 (30–42)	28 (24–33)	30 (26–37)	**<0.001**
**Marital status**				**<0.001 (23.048)**
Never married	10% (15)	4% (10)	7% (25)	
Married / engaged	58% (85)	40% (90)	47% (175)	
Separated / divorced	18% (26)	25% (57)	22% (83)	
Widowed	14% (21)	31% (70)	24% (91)	
**Education**				**<0.001 (17.916)**
None	18% (26)	23% (53)	21% (79)	
Elementary / primary	44% (64)	58 % (131)	52% (195)	
Secondary or higher	39% (57)	19% (43)	27% (100)	
**Drinks alcohol**	27% (39)	16% (37)	20% (76)	**0.017 (5.666)**
**Had any sex in the last six months**	45% (66)	34% (77)	38% (143)	**0.033 (4.552)**
**Had sex with regular partner in the last six months**	39% (57)	28% (63)	32% (120)	**0.026 (4.974)**
**Had sex with casual partner in the last six months**	7% (10)	7% (16)	7% (26)	0.927 (0.008)
**Knew HIV can be transmitted by having sex with another person**	96% (141)	95% (215)	95% (356)	0.595 (0.283)
**Knew consistent condom use prevent HIV transmission**	87% (128)	78% (178)	82% (306)	**0.034 (4.499)**
**Knew HIV cannot be transmitted via mosquito bites**	77% (113)	78% (176)	77% (289)	0.881 (0.022)
**Knew a HIV-infected person on ART may still infect their sexual partner**	90% (132)	86% (195)	87% (327)	0.267 (1.231)
**Knew ART medication can suppress HIV**	88% (129)	81% (183)	83% (312)	0.070 (3.288)
**Knew ART medication cannot get rid of HIV from the body**	70% (103)	77% (175)	74% (278)	0.129 (2.307)

^a^ Numbers in bold indicate that there is a statistically significant difference in the characteristic between males and females (p < 0.05).

^b^ Median (lower and upper quartile) and p-value from Mann–Whitney U test for difference in age between males and females were given for age. Five participants did not report their age.

Almost half the participants (47%) were married or engaged (of these, only three were engaged), about one-fifth were separated or divorced (22%), almost a quarter were widowed (24%) and a small proportion were never married (7%). Overall, 38% (95% CI: 33% - 43%) of participants had sexual intercourse, and 32% (95% CI: 27% - 37%) of participants had sexual intercourse with a regular partner in the last six months. Men were significantly more likely to be married or engaged (58%) compared with women (40%). Men were also more likely to have had sexual intercourse in the last six months with a regular partner of the opposite sex (39%, 95% CI: 31% - 47%) compared with women (28%, 95% CI: 22% - 34%). Of those who were engaged or married, 58% (95% CI: 51% - 66%) had sexual intercourse with a regular opposite-sex partner, whereas only 9% (95% CI: 5% - 14%) of those who were never married, separated or divorced had sex with a regular opposite sex partners (not shown in table). Small and similar proportions of men and women (both 7%) had sexual intercourse with casual opposite-sex partners in the last six months.

Data from the interviews reinforced the behavioural data on high levels of sexual abstinence. While reasons for sexual abstinence were not asked in the behavioural survey, in the interviews participants discussed a number of motivating factors for why they were not sexually active. These included social and individual responsibility to avoid further transmission of the virus, a fear of super infection and a lack of sexual desire since being diagnosed with HIV. Examples of these motivators are suggested in the following narratives from study participants:

I encourage other people not to dream about sex. You should abstain completely…It’s people living with the virus who decide whether this virus goes on or not. So if everyone is really honest and takes responsibility and abstain from sexual behaviours the virus will die with them. (*Woman living with HIV)*

What we should be preaching and telling people is don’t do it…I think that condoms are not the answer. The answer is no sex and that is it. *(Man living with HIV)*

Now that I am on medication I just don’t want to sleep with men. What if I give them my disease and they give me theirs? Like I will get double sickness or something. *(Woman living with HIV)*

Before I felt like having sex with anyone at anytime but now that I am on ART it has decreased my sexual drive. *(Man living with HIV)*

Abstinence from sexual intercourse was also the result of health education messages and the directives given from health care workers. These directives while posited within a type of positive living discourse entailed the incitement of fear and destruction. The following narrative examples illustrate this:

They told me not to eat greasy food and stopped me from having sex with men. Don’t get married…You can go and get married but you will destroy yourself again so don’t get married. *(Woman living with HIV)*

…when I go for trainings they say that the dirt is below and the clean water is on top and when you shake this water the dirt and the clean water will mix like when you have sex with a man the virus of the man and the woman will work hard and it will make you feel weak and symptoms will quickly appear and you will lose weight, feel very sick and die. That’s why my thoughts on not sleeping with a man is strong…I must stay by myself and the disease will just live in me without any strength. *(Woman living with HIV)*

Almost all participants reported that HIV can be sexually transmitted (95%), and over 80% knew that a HIV-positive person on ART may still infect their sexual partner (87%), that ART medication can reduce viral load (83%) and that consistent condom use prevents HIV transmission (82%). Comparatively fewer participants knew that HIV cannot be transmitted via mosquito bites (77%) and that ART medication cannot cure a person of HIV (74%). Significantly more men (compared with women) knew that consistent condom use prevents HIV transmission. Those with a higher level of education were more likely to know that HIV can be sexually transmitted (linear trend p = 0.002) and that HIV cannot be transmitted via mosquito bites (linear trend p < 0.001; not shown in table).

Table 2 shows the sexual behaviour of the 143 participants who had sexual intercourse in the last six months, of whom 66 were men and 77 were women. The majority of the 143 respondents had sex with a regular sexual partner only (77%), 7% had sex with both regular and casual sexual partners, 11% had sex with casual sexual partners only, and the rest (5%) comprised four women who did not report the type of sexual intercourse they had with their sex partner(s) and three men who only had sex with sex workers. There was no significant difference in consistent condom use amongst men and women during vaginal intercourse with either regular or casual sexual partners. Overall, 11 (17%) men reported that they had anal intercourse in the past six months, of whom one had anal intercourse with opposite-sex casual sexual partners only, one with both opposite-sex casual and regular sexual partners, four with opposite-sex regular sexual partners only and five had anal intercourse with same-sex casual sexual partners only. Only 11 (9%) women reported that they had anal intercourse with a regular sexual partner.

**Table 2:** Sex practice of the participants who had sex in the last six months

	**Male (n = 66)**	**Female (n = 77)**	**Overall (N = 143)**	**p (χ**^**2**^**) **^**c**^
**Had sex with regular partner**	86% (57)	81% (63)	84% (120)	0.461 (0.544)
***Consistent condom use during vaginal sex with regular partner ***^***a***^				0.088 (2.936)
Yes	54% (31)	39% (24)	46% (55)	
No	46% (26)	61% (38)	54% (64)	
***Condom use at last vaginal sex with regular partner ***^***a***^				0.085 (2.971)
Yes	70% (40)	55% (34)	62% (74)	
No	30% (17)	45% (28)	38% (45)	
***Had opposite-sex anal sex with regular partner ***^***b***^				0.887 (0.020)
Yes	9% (5)	10% (6)	9% (11)	
No	91% (52)	90% (57)	91% (109)	
**Had sex with casual partner**	15% (10)	21% (16)	18% (26)	0.384 (0.757)
***Consistent condom use during vaginal sex with casual partner***				0.646 ^c^
Yes	33% (2)	50% (8)	45% (10)	
No	67% (4)	50% (8)	55% (12)	
***Condom use at last vaginal sex with casual partner***				0.631 ^c^
Yes	67% (4)	62% (10)	64% (14)	
No	33% (2)	38% (6)	36% (8)	
***Had opposite-sex anal sex with casual partner***				0.538 ^c^
Yes	20% (2)	6% (1)	12% (3)	
No	80% (8)	94% (15)	89% (23)	
***Had same-sex anal sex with casual partner***				-
Yes	50% (5)	-	50% (5)	
No	50% (5)	-	50% (5)	
**Had any anal sex**	17% (11)	9% (7)	13% (18)	0.173 (1.854)

^a^ One female participant did not answer the question on frequency of condom use during vaginal sex with regular partner in the last six months.

^b^ As none had sex with same-sex regular partners in the last six months, only opposite-sex anal sex was reported with regular partner.

^c^ Pearson chi-square test was used for all variables except consistent condom use and having had opposite-sex anal sex with casual partner for which Fisher exact test was used.

Table 3 shows the factors associated with having had sexual intercourse in the last six months. Significantly fewer women had sex in the last six months in the unadjusted bivariate analysis, but this is not significant after adjusting for marital status in a Mantel-Haenszel test (OR_MH_ = 0.86; 95%CI = 0.53 - 1.39; χ_MH_^2^ = 0.37; p = 0.543; not shown in table) or for a combination of other factors in the logistic regression model. Those who were married, with a higher level of education, who drink alcohol, who were resident in the Southern region (i.e. Port Moresby) and who knew that HIV cannot be transmitted via mosquito bites were more likely to have had sexual intercourse in the last six months in both the bivariate and regression analyses.

**Table 3:** Factors associated with having had sex in the last six months (N = 374)

	**% (count)**					
	**Did not have sex**	**Had sex**	**Crude OR**	**p**^**a**^	**Adj. OR**	**p**^**a**^
	**(n = 231)**	**(n = 143)**	**(95% CI)**		**(95% CI)**	
**Female (versus Male)**	65% (150)	54% (77)	**0.63 (0.41, 0.96)**	**0.033**	1.03 (0.60, 1.77)	0.918
**Age **^**b**^				0.482		**-**
16-25	24% (55)	25% (36)	1.00		-	
26-35	45% (103)	50% (71)	1.05 (0.63, 1.77)	0.845	-	-
>35	30% (69)	25% (35)	0.77 (0.43, 1.39)	0.394	-	-
**Marital status**				**<0.001**		**<0.001**
Never married	9% (20)	4% (5)	**0.15 (0.05, 0.42)**	**<0.001**	**0.08 (0.02, 0.24)**	**<0.001**
Married / engaged	29% (66)	76% (109)	1.00		1.00	
Separated / divorced	27% (63)	14% (20)	**0.19 (0.11, 0.35)**	**<0.001**	**0.15 (0.08, 0.29)**	**<0.001**
Widowed	36% (82)	6% (9)	**0.66 (0.03, 0.14)**	**<0.001**	**0.05 (0.02, 0.12)**	**<0.001**
**Education**				**<0.001**^**c**^		**0.041**
None	25% (58)	15% (21)	1.00		1.00	
Elementary / primary	55% (128)	47% (67)	1.45 (0.81, 2.58)	0.213	1.20 (0.59, 2.42)	0.616
Secondary or higher	20% (45)	39% (55)	**3.37 (1.79, 6.38)**	**<0.001**	**2.43 (1.09, 5.41)**	**0.030**
**Region of residence**				0.064		**0.015**
Southern	25% (58)	34% (49)	1.00		1.00	
Highlands	67% (154)	62% (89)	0.68 (0.43, 1.09)	0.106	0.57 (0.31, 1.03)	0.063
Momase	8% (18)	4% (5)	**0.33 (0.11, 0.97)**	**0.034**	**0.18 (0.05, 0.62)**	**0.007**
**Drinks alcohol (versus Does not drink alcohol)**	16% (38)	27% (38)	**1.83 (1.10, 3.04)**	**0.020**	**2.04 (1.08, 3.87)**	**0.029**
**Knew HIV can be transmitted by having sex with another person**	95% (219)	96% (137)	1.25 (0.46, 3.42)	0.661	-	-
**Knew consistent condom use prevent HIV transmission**	81% (188)	83% (118)	1.08 (0.63, 1.86)	0.783	-	**-**
**Knew HIV cannot be transmitted via mosquito bites**	72% (166)	86% (123)	**2.41 (1.37, 4.22)**	**0.002**	**2.60 (1.31, 5.13)**	**0.006**
**Knew a HIV-infected person on ART may still infect their sexual partner**	85% (198)	90% (129)	1.54 (0.79, 2.99)	0.203	-	-
**Knew ART medication can suppress HIV**	83% (191)	85% (121)	1.15 (0.65, 2.03)	0.626	-	-
**Knew ART medication cannot get rid of HIV from the body**	71% (164)	80% (114)	1.61 (0.97, 2.65)	0.061	1.23 (0.66, 2.28)	0.509

^a^ Numbers in bold indicate that there is a statistically significant association with having sex in the last six months (p < 0.05).

^b^ Five participants did not report their age.

^c^ There was a significant linear trend (p < 0.001) of increase in having had sex in the last six months with higher education level with crude OR (95% CI) of 1.85 (1.37, 2.51).

Table 4 shows the factors associated with consistent condom use during vaginal intercourse with a regular sexual partner in the last six months. After adjustment for other factors, women were significantly less likely to use a condom compared with men. Having an HIV-negative regular sexual partner was associated with more consistent condom use, although this was not significant. This non-significance is possibly due to the lack of power as only a small number of participants (n = 26) identified that their regular sexual partner was HIV-negative (i.e. they were in a serodiscordant relationship). Almost all of the participants with a regular sexual partner had disclosed their HIV status to them (90%) and disclosure was significantly higher amongst those who reported they were married or engaged (94%) compared with those who were not married or engaged (67%; p = 0.003 from Fisher’s exact test). Disclosure of an HIV-positive status was also not significantly associated with consistent condom use. Of the 12 participants who did not disclose, one knew their partner was HIV-positive and another knew their partner was HIV-negative. Those who drank alcohol were more likely to consistently use a condom, compared with those who did not drink alcohol and those who knew that ART could not cure a person with HIV were over three times more likely to use a condom than those who did not in both the bivariate and regression analyses.

**Table 4:** Consistent condom use during vaginal sex with regular sex partner (N = 119)

	**% (count)**					
	**Inconsistent condom use**	**Consistent condom use**	**Crude OR**	**p**^**a**^	**Adj OR**	**p**^**a**^
	**(n = 64)**	**(n = 55)**	**(95% CI)**^**a**^		**(95% CI)**^**a**^	
**Female (versus Male)**	59% (38)	44% (24)	0.53 (0.25, 1.11)	0.088	**0.39 (0.17, 0.88)**	**0.023**
**Age **^**b**^				0.183		**-**
16-25	30% (19)	20% (11)	1.00		-	
26-35	51% (32)	47% (26)	1.40 (0.56, 3.50)	0.465	-	-
>35	19% (12)	33% (18)	2.59 (0.88, 7.63)	0.073	-	-
**Married/ engaged (versus Not married / engaged)**	91% (58)	78% (43)	0.37 (0.13 , 1.09)	0.060	0.34 (0.10, 1.13)	0.078
**HIV sero-concordance**				0.195		-
Concordant (i.e. partner is HIV positive)	64% (41)	56% (31)	1.00		-	
Disconcordant (i.e. partner is HIV negative)	16% (10)	29% (16)	2.12 (0.83, 5.39)	0.108	-	-
Unknown (i.e. partner’s status is unknown)	20% (13)	15% (8)	0.81 (0.30, 2.22)	0.687	-	-
**HIV positive status disclosed to partner**	92% (59)	87% (48)	0.58 (0.17, 1.97)	0.377	-	-
**Education**				0.110 ^c^		-
None	17% (11)	9% (5)	1.00		-	
Elementary / primary	50% (32)	40% (22)	1.51 (0.46, 5.03)	0.496	-	-
Secondary or higher	33% (21)	51% (28)	2.93 (0.85, 10.13)	0.074	-	-
**Region of residence**				0.751		-
Southern	33% (21)	27% (15)	1.00		-	
Highlands	63% (40)	69% (38)	1.33 (0.60, 2.97)	0.485	-	**-**
Momase	5% (3)	4% (2)	0.93 (0.14, 6.44)	0.944	-	**-**
**Drinks alcohol (versus Does not drink alcohol)**	16% (10)	35% (19)	**2.85 (1.16, 7.01)**	**0.017**	**2.76 (1.05, 7.21)**	**0.039**
**Knew HIV can be transmitted by having sex with another person**	94% (60)	98% (54)	3.60 (0.38, 33.98)	0.232	-	-
**Knew consistent condom use prevent HIV transmission**	84% (54)	82% (45)	0.83 (0.32, 2.19)	0.711	-	-
**Knew HIV cannot be transmitted via mosquito bites**	84% (54)	89% (49)	1.51 (0.51, 4.50)	0.454	-	-
**Knew a HIV-infected person on ART may still infect their sexual partner**	86% (55)	96% (53)	4.34 (0.87, 21.71)	0.051	4.88 (0.76, 31.27)	0.094
**Knew ART medication can suppress HIV**	84% (54)	89% (49)	1.51 (0.51, 4.50)	0.454	-	-
**Knew ART medication cannot cure HIV**	70% (45)	89% (49)	**3.45 (1.22, 9.71)**	**0.013**	**3.89 (1.30, 11.62)**	**0.015**

^a^ Numbers in bold indicate that there is a statistically significant association with consistent condom use during vaginal sex with regular sex partner (p < 0.05).

^b^ One participant did not report his age.

^c^ There was a significant linear trend (p = 0.038) of increase in consistent condom use with higher education level with crude OR (95% CI) of 1.74 (1.03, 2.94).

From the interviews it is evident that for people in sero-discordant sexual relationships, and where HIV status was disclosed, the use of condoms was something that was negotiated and entailed intimate partner communication. This was particularly the case for women with HIV. Women reported that they had initially started to use condoms with their regular HIV-negative partner but that as the relationship continued condom used discontinued. For example, one woman said the following about her HIV-negative husband:

‘He told me, I love you and we will live together. In my own belief your sickness will not get me’ he said…We stayed with condoms for six months but after six months he told me ‘How long are we going to use condoms? Will we use them until we die?’

There was no data in the qualitative interviews as to whether or not women would rather have continued using condoms but were unable to counter their husband’s preference. The qualitative interviews from married women and men whose partners were HIV-positive indicated that condoms were not needed as a result of concordant serostatus.

## Discussion

Overall, the level of sexual activity amongst the sample in this study was low. The overall percentage of both women and men on ART who reported sexual activity (38%) is much lower than the proportions found by some studies in developing country contexts see for example [28-32]. For example, the proportion of women in this study reporting sexual activity in the last six months is lower than that found among women in a three-country study of Brazil, South Africa and Uganda [5] where almost half had sexual intercourse in the last month (46%).

Not surprisingly there was a relationship between sexual intercourse in the past six months and a person’s marital status with married people more likely to have had sexual intercourse. Among those that have never married, were separated/divorced or widowed, 9% reported that they had sexual intercourse with a regular partner, but this is significantly less than those who reported themselves as married or engaged (58%). This finding on the relationship between sexual intercourse and marital status concurs with data from other developing countries with heterosexually driven epidemics. For example, married people with HIV in Kenya were significantly more likely to report sexual intercourse than those who were not [22]. Similarly, in Uganda, compared with HIV-positive people who were widowed, those who identified as married were also significantly more likely to be sexually active [16,17]. Likewise, a three-country study by Kaida et al. [5] showed that recent sexual activity for HIV-positive women in Brazil, South Africa and Uganda was significantly associated with being married.

Marital status was similarly important, although not statistically significant, in relation to consistent condom use. Those who reported not being married or engaged reported higher rates of consistent condom use compared with those who were married. HIV-positive people on ART who were married or engaged were more likely to be sexually active with a regular partner but were at the same time less likely to consistently use condoms, irrespective of a partner’s HIV status cf. [27].

The high level of sexual abstinence by women in this study suggests that although the literature to date has shown little to no control by women over their sexual and reproductive lives, the women in this study, and in particular those who were not married, were not devoid of power to control their sexual life as is indicated elsewhere. And merely because a woman with HIV does not use a condom with her husband does not automatically and unequivocally equate with disempowerment, as suggested by the qualitative interviews. The qualitative data from this study offers some insights into this particular aspect of the behavioural data and involves intangible issues associated with long-term relationships, intimate communication and love. Rather, condom use is negotiated in light of knowledge of a partner’s HIV status, long-term commitment and love. For those women with HIV-negative partners inconsistent condom use appears to be the initiated by their regular male partners. As one of the quotes used in the Results section of this paper illustrates, it appears that their regular partners (husbands) had accepted their HIV status and were not afraid of becoming infected. International research supports this inference, especially research in serodiscordant couples where it has been shown that the HIV-negative partner has been the principal initiator of unsafe sex [33,34]. One reading of this could be that men had a poor understanding of their risk for HIV acquisition. Another reading of the data is that women were not able to counter their partners’ wishes to discontinue using condoms. A more likely scenario from an detailed reading of that data suggests that this absence of fear was based on elusive aspects of a relationships including acceptance of HIV, understanding and experience of risk and other relationship issues such as duration of the relationship, love and intimacy [27]. Furthermore, what it means to be in a serodiscordant relationship is context-specific and it cannot then be assumed that risk-management and decisions about sexual practices will be and indeed should be the same globally [35].

Women were not questioned about lifetime sexual or physical violence or about sexual relationship power, so we cannot therefore postulate about their ability to have control in their sexual and reproductive lives more generally, as is possible in other studies cf. [36]. There is however some research in PNG that does link HIV status with lifetime intimate partner violence [37]. Unlike other studies that link HIV risk to intimate partner violence amongst newly diagnosed women with HIV, this study was of women who were already diagnosed with HIV. Furthermore, of the women with HIV-negative partners, all were in relationships that had been initiated following their diagnosis and therefore we cannot comment of the relationship between violence and HIV risk as others have done.

It is true that in PNG women are subjugated to much gender-based violence and have been reported to have minimal to no control over their sexual and reproductive lives [38,39]. Furthermore, international research has consistently reported that women’s vulnerability to HIV is linked to an absence of power, ability to negotiate sexual relationships and experiences of intimate partner violence cf. [36,40]. Moreover, it has long been posited that gender-based power differentials between women and men often compromises a woman’s ability to negotiate condoms cf. [41,42]. Although it may well be possible that women in this study were unable to counter their HIV-negative partners’ desire to discontinue using condoms, this is not what the data suggested. This is an interesting finding and warrants further scrutiny especially in light of the long history of research on gender, HIV and vulnerably that suggests otherwise. It would however appear that it is possible that this data contributes to a recent and increasing recognition within PNG that women can and indeed are exhibiting agency in choosing sexual and lifetime partners [43], with some becoming involved in polygamous marriages in the desire to obtain status through money and items of modernity such as cars and expensive mobile phones [44].

In countries where viral load testing is part of routine clinical care and management, informed choices about viral suppression and risk of HIV transmission are possible. To date this is not the situation in PNG. And although in other settings there is an awareness of the low risk of sexual transmission while on ART (for example the Swiss Statement) there is no such discourse or health messaging in PNG. In fact, there is evidence in PNG that PLHIV whose partner is also positive are recommended to still use condoms for fear of ‘super-infection’ [45]. Therefore, it is unlikely that such knowledge about viral load and infectivity can account for unprotected sex between sero-discordant partners for whom condoms play a greater role than where both partners are HIV-positive.

Although people living with HIV are increasingly making active choices to have children as a recent qualitative study of prevention of mother-to-child transmission in PNG has found [45], it is our informed opinion in this study that the low use of condoms was not reflective of a desire on behalf of HIV-positive women to conceive or of HIV-positive men to father a child. In 2008, when this study was conducted, ART was not widely available, and there was little to no discussion of HIV-positive women actively choosing to reproduce (as opposed to testing HIV-positive while pregnant) and prevention of mother to child transmission programs were still in their infancy. No women in the qualitative arm of our study reported wanting to have children after being diagnosed with HIV and neither did any men report wanting to have children. Furthermore, the qualitative data suggests that we can be confident that participants’ low rates of reported sexual activity was not an artefact of social desirability or indeed a discrepancy in what is constituted as sexual intercourse. Rather the qualitative data suggests that in the early stages of ART availability in PNG, many PLHIV were against the notion of both themselves and any other PLHIV from having sex. At the time of this study participants were reporting that reasons for sexual abstinence included a personal and social responsibility not to transmit HIV to another person and a fear of becoming re-infected with HIV. There was no gender difference in reported reasons for sexual abstinence. Additionally, the role of others, particularly health care workers, influencing a person’s beliefs and therefore decisions about sexual behaviour were also deemed important. This is not surprising since adherence to ART was also related to following instruction from the doctor/health care worker [46]. As people stay on treatment and as people live longer it will be important to chart if and how sexual behaviour changes and if the meanings ascribed to sexual intercourse and condom use similarly alter.

Since much of the HIV prevention message in PNG focuses on reducing extra-marital relationships and being faithful to one’s partner, it appears that safe sex within marriages of people living with HIV may need to be emphasised. Condoms have traditionally been seen in PNG as something used outside of a marriage; sex within a marriage is perceived to be safe and this is suggested by the ‘one faithful partner concept’ [47-50]. However in this study of HIV-positive people, those who were married or engaged were proportionally more likely to report having an HIV-positive partner than those who were not, and for those with HIV-positive partners, the need for consistent condom use to prevent transmission is lessened [48]. Though not statistically significant, proportionally less of those with a HIV-positive partner (43%) or of those who did not know the status of their regular partner (38%) consistently used condoms compared with those whose partner was HIV-negative (62%). In other words, consistent condom use was much higher (OR 2.12) among participants whose partners were known to be HIV-negative. Others have similarly identified that the HIV status of one’s partner affects condom use [27,51].

Almost all of the participants with a regular sexual partner had disclosed their HIV status to them and disclosure was significantly higher amongst those who were married or engaged compared with those who were not. This rate of disclosure to a sexual partner was much higher than that found in other studies, which report disclosure rates to regular partners ranging between 62 - 80% [52-55]. Similarly this data suggests that compared with positive people in other settings who report barriers to disclosure of serostatus cf. [56], people in PNG experienced fewer obstacles in the disclosure of HIV in intimate sexual relationships.

Consistent use of condoms was not significantly associated with HIV knowledge. Knowledge about the preventative nature of condoms against the sexual transmission of HIV was high (77%), indicating, as others have found, that there was a discrepancy between knowledge and behaviour. Among those that reported vaginal intercourse in the last six months, less than half (46%) reported consistent condom use. However, knowing that ART cannot cure a person of HIV was significantly associated with consistent condom use (OR 3.89). In other words, people with living HIV who were well-informed about their ART were using condoms more consistently. This contrasts with South African data which found that HIV and ART knowledge was not associated with condom use [19]. The association between education level and knowledge that HIV can be transmitted via sexual intercourse, but not via mosquito bites indicates that there could be some confusion about transmission of HIV which may be different from other commonly known blood-borne pathogens such as malaria amongst people who are less educated. This suggests that educational materials targeting this confusion may be beneficial.

Our study found that there was a strong association between having sexual intercourse in the last six months and consuming alcohol. On the other hand, there was a strong association between consistent condom use and consuming alcohol. While other behavioural studies have shown a relationship amongst HIV-positive people between drinking alcohol and high risk sexual behaviour [14,32,57,58] this was not the case in this study of Papua New Guineans on ART.

## Conclusions

Given that inconsistent condom use is more frequent in married and engaged relationships and in relationships where HIV status is undisclosed, structural prevention efforts that prioritise and increase access to couple testing and support safe HIV disclosure are likely to play an important role in the prevention of intimate partner transmission in PNG. As people’s health continues to improve on ART it may well be that people currently single or widowed may re-engage in sexual relationships and will need support and education to do so in a safe manner. Results from this study show that people living with HIV who are well-informed about ART are more consistent in their use of condoms. It thus appears that health care services play an important role as a structural facilitator for HIV prevention cf. [59] where health education during a person’s preparation with a health care worker to initiate ART plays an important role in the prevention of HIV through the use of condoms. Therefore, positive prevention needs to take a holistic view to preventing secondary transmission of HIV through wider HIV education such as ART rather than messages that simply chant to always use a condom. It also reinforces the wider health benefits of access to ART and good clinical education about the use of ART. Continuing to engage people with HIV in positive prevention, of discussing serostatus mixing and HIV disclosure will be important in preventing the secondary transmission of HIV in PNG and are likely to play a far greater role than a one-dimensional HIV prevention message of always using a condom. Moreover, as greater numbers of people are diagnosed with HIV; HIV-positive people are prescribed ART; survival rates increase and biomedical markers such as CD4 count and possibly viral load become part of standard clinical practice then, the sexual lives of PLHIV in PNG are likely to have changed and further research is warranted.

## Methods

The data were collected as part of a mixed-method study undertaken in six provinces throughout PNG between February and July 2008. The purpose of the study was to explore the social experience of HIV-positive men and women on ART.

A total of 374 HIV-positive people over the age of 16 and had been on ART for more than two weeks were recruited using a non-probability, convenience sampling methodology. As of December 2007, just prior to the commencement of this study there were 2,250 people enrolled on ART (185 of whom were children [60]). This survey therefore accounted for around 18% of adults on ART. Participants were recruited through a number of sites including ART prescribing sites, PLHIV drop-in centres and support groups in six provinces: Southern Highlands Province, National Capital District, Eastern Highlands Province, Morobe Province, Chimbu Province and Western Highlands Province.

Participants were invited by a third party (other PLHIV or health care workers) to participate in the study. All potential participants were explicitly informed that they were under no obligation to participate, and their access to ART or any form of treatment or support would not be affected should they decide not to participate in the study. Health care workers often gave a large ‘toksave’ (public notice) to PLHIV at the ART clinic waiting to be seen by a health care worker rather than individually inviting participants to participate.

After potential participants provided initial consent, they were directed to a nominated researcher. Once they had made contact, the researcher explained the purpose of the study and outlined what participation would entail. Upon providing informed consent, the participant completed an interviewer-administered questionnaire in either *Tok Pisin*, a PNG lingua franca, or English.

The interviewers were Papua New Guineans undergoing an intensive HIV social research training cadetship, coordinated by the PNG Institute of Medical Research and the University of New South Wales. In addition, a person living with HIV was employed as a member of the research team and trained in the methodologies of the study. Analysis of the data was undertaken by the lead authors.

The survey included items on socio-demographics, knowledge and beliefs around HIV and treatment, stigma and discrimination, health and well-being, disclosure, food security, alcohol use, ART adherence, sexual practices, and access to services. Standardised scales were used and piloted prior to study. At the end of the questionnaire one qualitative question on the meaning of ART in people’s lives was also asked.

No names were recorded on the questionnaire. All questionnaires were coded by province and recruitment site. A sub-sample of 36 participants also participated in a semi-structured qualitative interview. Qualitative data was used to triangulate behavioural data. The results reinforced key quantitative findings and provided socio-cultural and gendered context to the meaning of the behavioural data.

### Ethical considerations

Ethics approval for this study was granted by the Papua New Guinea Medical Research Advisory Committee, the Research Advisory Committee of the National AIDS Council Secretariat of Papua New Guinea, the Papua New Guinea Institute of Medical Research Internal Review Board and the Human Ethics Committee of the University of New South Wales.

### Questions and variables on sex practice

All participants were asked whether they had sexual intercourse in the last six months. Those participants who had sexual intercourse in the last six months were then asked whether they had sexual intercourse with a regular and/or a casual opposite sex partner in the last six months and whether they had vaginal and/or anal intercourse with these partners. Men were asked an additional question on whether or not they had sexual intercourse with another man in the past six months. Of those who had vaginal intercourse in the last six months with a regular or casual sex partner, participants were asked whether they never used a condom, or sometimes, almost every time or always used a condom during vaginal intercourse. Consistent condom use during vaginal intercourse was dichotomised into “yes” for using a condom every time versus “no” for all other answers (i.e. never, sometimes, almost every time or no response). Participants were also asked whether they used a condom during the last vaginal or anal intercourse with their regular or casual sex partner.

### Statistical analysis

All categorical variables were analysed using the χ2 test for bivariate association. All continuous variables were analysed using the Mann–Whitney U test. Variables with p < 0.10 in the bivariate analyses were entered into a logistic regression model to estimate adjusted odds ratios and 95% confidence intervals for further examining factors associated with sexual practice and condom use with regular partners. As none of the men had anal intercourse with a regular male partner in the last six months, the analyses on intercourse and condom use with regular sex partners comprises only regular partners of the opposite-sex. All analyses were carried out using Stata v11 [61].

### Qualitative analysis

All interviews were digitally recorded, transcribed and translated into English. All identifiable information was removed. All interviews were managed and analysed using Nvivo 9 qualitative analysis software. Analysis of interviews was based on a grounded theory approach to data [62]. After initial reading of all of the interviews, memo writing and data comparison, a codebook was developed by two of the researchers. Interviews were double coded and where a discrepancy emerged the two researchers discussed the data and a decision on the final code made. Data saturation was reached with no new themes emerging. Preliminary data analysis was presented at team meetings and discussed during the writing up of the study report and subsequent publications.

## Competing interests

The authors declare that they have no competing interests.

## Authors’ contributions

WYNM led the analysis of quantitative data and drafting of the paper. AK designed the study, secured funding, oversaw data collection and data analysis, led the analysis of qualitative data, contributed to the drafting of the manuscript and revising it critically for important intellectual content and gave final approval of the version to be submitted. AF oversaw the first stages of data analysis of the study and contributed to the drafting of the manuscript. PS contributed to the drafting of the manuscript. HW assisted in securing funding for the study and overseeing the implementation of it and contributed to the drafting of the manuscript. MK, AM, BK, RE, FA, BC, LW, LP and SN collected data and participated in data analysis for the manuscript. TL assisted in data analysis for this manuscript. PMS assisted in securing funding for the study, oversaw the implementation of the study and contributed to the drafting of the manuscript. All authors read and approved the final manuscript.
